# The ZorO-OrzO type I toxin–antitoxin locus: repression by the OrzO antitoxin

**DOI:** 10.1093/nar/gkt1018

**Published:** 2013-11-06

**Authors:** Jia Wen, Daniel Won, Elizabeth M. Fozo

**Affiliations:** Department of Microbiology, University of Tennessee, Knoxville, TN 37996, USA

## Abstract

Type I toxin–antitoxin loci consist of two genes: a small, hydrophobic, potentially toxic protein, and a small RNA (sRNA) antitoxin. The sRNA represses toxin gene expression by base pairing to the toxin mRNA. A previous bioinformatics search predicted a duplicated type I locus within *Escherichia coli* O157:H7 (EHEC), which we have named the gene pairs *zorO-orzO* and *zorP-orzP*. We show that overproduction of the *zorO* gene is toxic to *E. coli*; co-expression of the sRNA OrzO can neutralize this toxicity, confirming that the *zorO-orzO* pair is a true type I toxin–antitoxin locus. However, OrzO is unable to repress *zorO* in a strain deleted for RNase III, indicating that repression requires cleavage of the target mRNA. Sequence analysis and mutagenesis studies have elucidated a nucleotide sequence region (V1) that allows differential recognition of the *zorO* mRNA by OrzO and not OrzP, and a specific single nucleotide within the V1 of OrzO that is critical for repression of *zorO*. Although there are 18 nt of complementarity between the OrzO sRNA and the *zorO* mRNA, not all base pairing interactions are needed for repression; however, the amount needed is dependent on whether there is continuous or discontinuous complementarity to the target mRNA.

## INTRODUCTION

As a newly recognized class of gene expression regulators, small noncoding RNAs have been found in all kingdoms of life. In bacteria, these RNAs are referred to as small RNAs (sRNAs) and are often 50–300 nt in length. Some sRNAs bind to proteins and modulate their functions, as exemplified by the CsrB RNA of *Escherichia coli*, which directly binds and sequesters the CsrA protein, thus affecting carbon metabolism in *E. coli* (1). However, the majority of characterized sRNAs function by base pairing to target mRNAs, either regulating the translation or altering the stabilities of the targets.

These base pairing sRNAs fall into two categories: *trans*-encoded sRNAs and antisense sRNAs. *Trans*-encoded sRNAs are located at chromosomal positions distal from the genes they regulate. These sRNAs typically interact with their targets by limited base pairing and often require the RNA chaperone Hfq to function [reviewed in (2)]. True antisense sRNAs are encoded on the opposite strand of DNA to their targets. They share extensive base pairing potential (usually ≥60 nt) to their target mRNAs [reviewed in (3,4)]. Although antisense sRNAs were initially discovered on mobile genetic elements, several pairs have been described on bacterial chromosomes [reviewed in (5)].

A subset of antisense sRNAs in bacteria can repress the expression of small proteins <60 amino acids in length. These proteins are usually hydrophobic and toxic when overproduced. The sRNA (antitoxin) base pairs with the toxin mRNA and affects the translation and/or the stability of the mRNA. Gene pairs consisting of a toxic protein-encoding gene and a corresponding antitoxin sRNA-encoding gene are referred to as type I toxin–antitoxin systems. The first type I pair to be identified was the *hok/sok* locus of the R1 plasmid in *E. coli*, whereby Hok is the toxin, and Sok the antitoxin (6). The *hok* mRNA has a long half-life, whereas the Sok sRNA has a short half-life. When cell division occurs, a daughter cell that has not inherited the R1 plasmid will die owing to the quick degradation of the Sok sRNA and the translation of the more stable *hok* mRNA. Besides the *hok/sok* locus, several type I toxin–antitoxin gene pairs have been described on other plasmids in gram negative bacteria and were also shown to play a role in plasmid maintenance [reviewed in (7)]. Additionally, the RNAI-RNAII locus of the pAD1 plasmid of *Enterococcus faecalis* clearly showed that similar loci were present in gram positive bacteria (8–10). Homologs of these plasmid loci were later found within bacterial chromosomes (11–14).

In recent years, several type I toxin–antitoxin systems that do not have homology to plasmid sequences have been identified and characterized in a number of bacterial chromosomes. Although both the toxin and antitoxin-encoding genes are located at the same locus, their relative positioning varies among the chromosomally encoded type I pairs. Some antitoxins overlap with the 5′ untranslated region (UTR) of their targets, such as the Rdl sRNAs of the Ldr-Rdl family in *E. coli* (15), or the 3′ UTR, like RatA and SR4 of the TxpA-RatA and BsrG/SR4 loci of *Bacillus subtilis* (16,17). Others directly overlap with the coding sequences of the target mRNAs, as exemplified by the Ibs-Sib family of *E. coli* in which Sib sRNA completely overlaps with the *ibs* mRNA coding region (18).

Along with these, there are a few unconventional type I loci in which the antitoxins are encoded divergent from the toxin-encoding genes [reviewed in (19)]. Unlike the traditional type I loci that can have ≥60 nt base paring potential, the unconventional type I loci have limited base pairing, often only 18–21 nt. These loci include the TisAB-IstR and the ShoB-OhsC pairs in *E. coli* (18,20).

**Figure 1.**
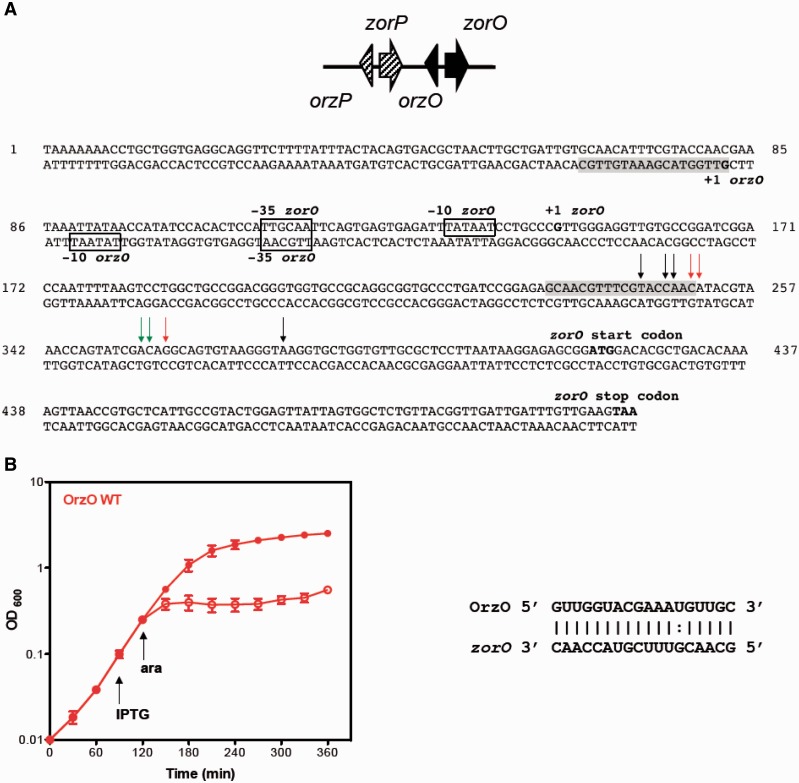
The *zorO-orzO* gene locus is a true type I toxin–antitoxin system. (**A**) Genetic organization of the duplicated *zor-orz* locus in *E. coli* EDL933. Sequence details of the *zorO-orzO* genes; region of base pairing is shaded, and previously mapped transcription starts are indicated in bold (14). Predicted −35 and −10 promoter elements are boxed. Black arrows indicate RNA processing products found in both the wild type and *Δrnc* strain; green arrows, wild type strain only; red arrows, *Δrnc* strain only. (**B**) Overexpression of OrzO can repress *zorO*-mediated toxicity. *E. coli* strain UTK007 (see Supplementary Table S1 for details) was transformed with pBR-plac-o*rzO* (induced by IPTG) and pBAD-*zorO* (induced by arabinose). The strain was grown to OD_600_ ≈ 0.1, and the culture was split into two. IPTG (1 mM final concentration) was added to one half of the culture. Arabinose (0.2% final concentration) was added 30 min later to each culture and OD_600_ was measured over time. Solid symbols indicate the addition of IPTG; open symbols, no IPTG. Shown are the mean values ± standard deviations for three independent cultures. Predicted region of base pairing is shown.

A previous bioinformatics search identified two highly homologous loci encoded in tandem in the chromosome of *E. coli* O157:H7 EDL933 (EHEC) (14). Each locus includes two genes: one encodes a small hydrophobic protein, and the other encodes an sRNA that is located divergently from the protein-encoding gene. This genetic organization is similar to that of the type I toxin–antitoxin pairs ShoB-OhsC and TisAB-IstR. In the two newly identified loci, the small protein-encoding genes (29 amino acids) were previously annotated as *z3289* and *z3290*. We have since renamed *z3289* and *z3290* as *zorO* (Z protein often repeated) and *zorP*, respectively. Additionally, we have denoted the sRNA gene divergent from *zorO* as *orzO* (overexpression represses *zor* toxicity) and the sRNA gene divergent from *zorP* as *orzP* (Figure 1A). The sRNAs contain regions of complementarity (18–19 nt) to the mRNAs, suggesting that they could base pair. In addition, the *zor-orz* loci are highly conserved in most commensal and pathogenic *E. coli* and *Shigella* strains but not in the common lab strains, like *E. coli* MG1655 (14).

Overexpression of ZorO was shown to be toxic; however, it was never demonstrated that the sRNA OrzO could repress *zorO*-induced toxicity (14). Thus, is *zorO-orzO*, a true type I toxin–antitoxin locus? Given the high homology between the *zorO-orzO* and *zorP-orzP* loci, is there ‘cross-talk’ in the regulation? In this study, we confirmed that the *zorO-orzO* locus is indeed a true type I toxin–antitoxin system. Using the inherent toxicity associated with *zorO* overproduction, we performed mutational analyses to further investigate the requirements for successful repression by the OrzO antitoxin. Through these analyses, we revealed that the 5′ end of the OrzO sRNA can solely repress *zorO* and the V1 region within 5′ end of the Orz sRNA determines the specific recognition of the *zor* target.

## MATERIALS AND METHODS

### Bacteria strains and plasmids

All bacterial strains and plasmids used in this work are listed in Supplementary Table S1. The sequences of all oligonucleotides are listed in Supplementary Table S2.

### Growth conditions

*Escherichia coli* strains were grown at 37°C in Luria-Bertani (LB) medium with shaking. Antibiotics were added when necessary at the following concentration: 100 μg/ml ampicillin, 25 μg/ml chloramphenicol, 25 μg/ml kanamycin. Arabinose was added to a final concentration of 0.2 or 0.002% as indicated. Isopropyl β-D-1-thiogalactopyranoside (IPTG), when used, was at a final concentration of either 0.1 or 1 mM, as indicated.

### Generation of strains for rescue experiments

All strains used in the described rescue experiments are derived from DJ624, which contains *ΔlacX74* and does not possess the *zor-orz* locus (21). To better control expression from the P_BAD_ promoter across the population of cells, the *araE* gene was placed under control of the P_CP18_ constitutive promoter through P1 transduction of the *kan*-P_CP18_-*araE* allele (22). The kanamycin cassette was then removed using pCP20, resulting in strain UTK007 (23). Generation of the *hfq* and *rnc* deletions was performed using the mini-λ-Red recombination system (24–26). In each case, pKD4 served as a polymerase chain reaction (PCR) template for amplifying the kanamycin cassette using primers containing 20 nt of homology to the kanamycin cassette, flanked by ∼40 nt of homology to the chromosomal region (25).

### Plasmid construction

For overexpression of the sRNAs OrzO, OrzP, OhsC, IstR, the genes were amplified from genomic DNA (*E. coli* O157:H7 EDL933) and ligated into the AatII and EcoRI sites of pBR-plac generating pBR-plac-*orzO,* pBR-plac-*orzP* pBR-plac-*ohsC* and pBR-plac-*istR* (21). The *zorO* gene was ligated into the PstI and HindIII sites of pEF21 from the mapped +1 of its transcription, generating pEF21-*zorO* (pBAD-*zorO* in the text) (14,18). Mutations of individual nucleotide residues for all constructs were performed by site-directed mutagenesis of pBR-plac-*orzO*, pBR-plac-*orzP* or pEF21-*zorO*. For generation of pBR-pLac-*orzO* 6(1)10(1) and pBR-plac-*orzO* (1)5(1)11, pBR-plac-*orzO* 6(1)11 served as the template. The two chimeric constructs, pBR-plac-*orzO-ohsC* and pBR-plac-*orzO-istR*, were generated using SOE PCR (27,28). PCR product A amplified the first 18 nt of *orzO* from pBR-plac-*orzO* along with 100 nt upstream of vector sequence (including the AatII cut site); the 3′ end of the product contained 15 nt of PCR product B. PCR product B amplified either *ohsC* or *istR* from *E. coli* O157:H7 EDL933 genomic DNA; this product began immediately following the region of base pairing that these RNAs would have for their respective targets (18,20). The 5′ end of PCR B contained 15 nt of homology to the 5′ end of *orzO*. The products were then spliced together via PCR, digested with AatII and EcoRI and ligated into pBR-plac. Plasmid DNA was isolated using the Qiagen Mini Plasmid Kit; PCR products were isolated using the Qiagen PCR Purification Kit.

### Rescue experiments

Rescue experiments were as described previously (15,18). The pBR-plac plasmid carrying the *orz* wild type/mutant gene under an IPTG-induced P_LlacO-1_ promoter was initially transformed into UTK007 or one of the derivatives described above, through electroporation (21). Afterward, the pEF21 plasmid containing the *zor* wild type/mutant gene under an arabinose-induced P_BAD_ promoter was transformed into the same cells (29). The resulting transformants harboring two plasmids were grown overnight and then diluted to OD_600_ of 0.01. When the OD_600_ reached ≈ 0.1, the cultures were split and IPTG was added to half the culture to a final concentration of either 0.1 or 1 mM for the overexpression of the *orz* gene. Thirty minutes after IPTG induction, arabinose was added to a final concentration of either 0.002 or 0.2% for the overproduction of the *zor* gene. OD_600_ was taken and recorded every 30 min. Shown are averages ± standard deviations for a minimum of three independent experiments.

### RNA extraction

To examine RNA levels of *zorO* and OrzO, cells harboring the appropriate plasmids were grown to an OD_600_ ≈ 0.2, and arabinose was added as indicated to induce *zorO* (time 0). Five minutes after induction with arabinose, the culture was split and IPTG (0.1 or 1 mM) was added to half. RNA was extracted via direct lysis as previously described with some modifications (30). Cells (750 μl aliquots) from time 0, 5, 15, 30 and 60 min after arabinose were harvested and incubated at 65°C with 500 μl acid phenol: chloroform (preheated to 65°C) and 102 μl of direct lysis solution (320 mM sodium acetate, 8% SDS, 16 mM EDTA) for 10 min. After incubation, the mixtures were centrifuged at 13 000 rpm at room temperature for 10 min. Supernatants were transferred to tubes containing 500 μl of 65°C phenol: chloroform, mixed thoroughly and spun again for 10 min at room temperature. The supernatants were transferred and extracted twice with 400 μl phenol: chloroform. The RNA was then ethanol precipitated and resuspended in RNase-free water. When needed, RNA was treated with Turbo DNaseI (Life Technologies) following manufacturer’s instructions.

### Northern analysis

Total RNA (10 μg) isolated at indicated time points following arabinose induction was separated on a denatured 6 (for the separation of *zor* mRNA) or 8% (for the separation of *orz* sRNA) polyacrylamide-urea gel and transferred to a Zeta-Probe Genomic GT membrane (Bio-Rad). Specific probes were generated by end-labeling oligonucleotides with γ-^32^P by T4 polynucleotide kinase. Incubation of the membrane and washes were performed as outlined by Opdyke *et al.* (31). Northern analysis was performed from a minimum of three independent experiments for every construct examined.

### Quantification of RNA levels

The relative intensities of the resulting bands from northern analysis detected via autoradiography were quantified using ImageJ (32). The intensity of the band for WT at T_15_ (induced with the level of IPTG indicated) was set to a value of ‘1’ as a standard for comparison. Relative intensities to WT at T_15_ for the other bands on the same northern blot were thus calculated. Averages and standard deviations were determined, and the statistical significance was calculated using Student’s *t*-test.

### Primer extension

Total RNA (5 μg) isolated 30 min after arabinose induction from rescue experiments was separated on a denatured 8% polyacrylamide-urea gel as described previously (14,31).

## RESULTS

### *zorO-orzO* is a bona fide type I toxin–antitoxin locus

The *zorO* gene of *E. coli* O157:H7 EDL933, initially annotated as *z3289*, was predicted to be type I toxin (14). Overexpression of the small protein from a high-copy plasmid in *E. coli* MG1655 led to cell growth stasis as well as a decrease in colony forming units, indicating that ZorO is toxic (14). In the same study, the sRNA OrzO (denoted at that time as sRNA-1) was detected via northern analysis. This sRNA, though encoded divergent from the toxin gene, could potentially base pair with the 5′ UTR of the *zorO* mRNA (Figure 1A and B).

Despite these data, the crucial question remained whether the *zorO-orzO* pair is a true type I toxin–antitoxin locus. If so, then the OrzO sRNA should be able to repress the toxicity of ZorO overproduction. Rescue experiments were used to test if OrzO can repress ZorO. We expressed *zorO* from an arabinose-inducible promoter (P_BAD_) on one plasmid and *orzO* from an IPTG-inducible promoter (P_LlacO-1_) on a second compatible plasmid (15,18). These experiments were performed in a strain derived from *E. coli* MG1655 that does not possess the *zor-orz* loci so as to eliminate any possible repression from a chromosomally encoded OrzO. While the induction of *zorO* led to cell growth stasis, indicating that ZorO is toxic, the co-expression of OrzO prevented this cell stasis, suggesting that OrzO serves as an antitoxin for ZorO (Figure 1B). Thus, we conclude that the *zorO-orzO* pair is a true type I toxin–antitoxin locus.

### RNase III but not Hfq is required for repression of *zorO*-induced toxicity

The majority of *trans*-encoded sRNAs characterized to date require the RNA chaperone Hfq to function efficiently, whereas the antisense sRNAs typically do not (2,7,33). However, given that there is limited complementarity between the *zorO* mRNA and the corresponding OrzO sRNA (18 nt perfect complementarity), we wanted to examine whether Hfq is required for the observed repression of the ZorO toxin. A similar rescue experiment was performed as outlined above in a strain deleted for *hfq*. The results showed that the OrzO sRNA can fully repress *zorO* in both the wild type and the *Δhfq* strain (Supplemental Figure S1), indicating that Hfq is not needed for the OrzO sRNA to regulate *zorO*.

**Figure 2.**
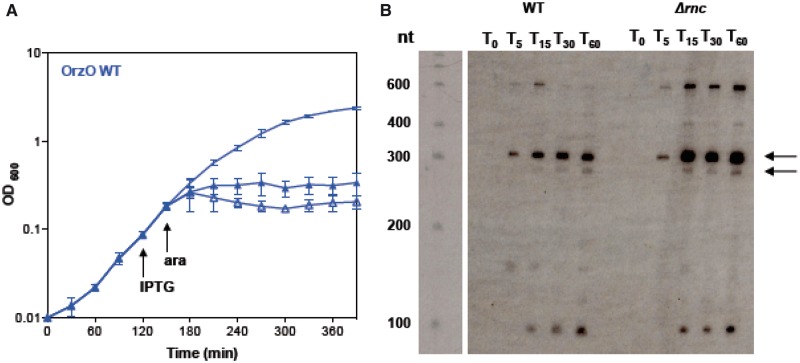
RNase III is critical for OrzO repression of *zorO.* (**A**) Overexpression of OrzO cannot repress *zorO*-mediated toxicity in a strain deleted for *rnc*. *E. coli* strain UTK011, which is deleted for *rnc,* was transformed with pBR-plac-*orzO* and pBAD-*zorO*. The strain was grown to OD_600_ ≈ 0.1, and split into three. One of the three cultures received no supplementation. IPTG (1 mM) was added as indicated to one culture. After 30 min, arabinose (0.2%) was added to two of the cultures and cell growth was monitored by measuring the OD_600_ over time. Solid symbols indicate the addition of arabinose and IPTG; open symbols, arabinose and no IPTG; no symbol indicates unsupplemented culture. Shown are the averages ± standard deviations from three independent experiments. (**B**) Accumulation of full-length *zorO* mRNA in the *rnc* deletion strain. Total RNA was isolated from a wild-type strain (UTK007) and an *rnc* deletion strain (UTK011) harboring pBAD-*zorO* 0, 5, 15, 30, 60 min after the addition of 0.2% arabinose. Arrows indicate the predicted full-length *zorO* transcripts.

RNase III, a double-stranded ribonuclease, has been shown to be critical for cleaving paired toxin–antitoxin complexes including *hok/sok*, *tisB-istR*, *bsrG/sr4*, *txpA-ratA*, as well as for the sRNA GadY and its mRNA target *gadX-gadW* (16,20,34–36). To determine whether repression by OrzO was dependent on expression of RNase III, we performed our rescue experiment in a strain deleted for *rnc*, the gene encoding RNase III. The results (Figure 2A) show that OrzO is incapable of rescuing *zorO*-induced toxicity in this mutant, even at low levels of inducing agent (0.002% arabinose, see below; data not shown). When *zorO* is expressed with OrzO in a wild-type strain, we detect major transcripts of ∼280 and 310 nt in length via northern analysis (Figure 2B). When examining *zorO* in the *Δrnc* strain, we noted an accumulation of these full-length transcripts in comparison with expression in the wild-type strain, suggesting that the full-length mRNAs are more stable in the deletion strain (Figure 2B). This implies that cleavage of the *zorO*-OrzO RNA duplex by RNase III is important for repression of *zorO-*induced toxicity. Surprisingly, OrzO levels (induced by 1 mM IPTG) appear reduced in the *Δrnc* strain (Supplementary Figure S2A).

We attempted to map the cleavage sites of *zorO* when OrzO is expressed in a wild type and a *Δrnc* strain using primer extension. Similar to our northern analyses, we noted an accumulation of full-length *zorO* mRNA in the *Δrnc* strain even when OrzO is expressed (Supplementary Figure S2B). However, we are able to map OrzO-specific cleavage sites within the *zorO* mRNA in the *Δrnc* strain (Figure 1A, Supplementary Figure S2B), similar to what has been reported for cleavage of *gadX-gadW* by GadY and cleavage of *cII-O* by the OPP RNA (35,37,38). This finding suggests that while RNase III may be critical for regulation of *zorO* repression, there may be additional factors involved (see ‘Discussion’ section). As summarized in Figure 1A and shown in Supplementary Figure S2B, the major processing sites lie within the region of base pairing for both the wild type and *Δrnc* strain. We did note that in the *Δrnc* strain, there were sites that appear to be shifted from the wild-type cleavage sites, as well as decreased cleavage at some sites (Figure 1A, Supplementary Figure S2B). We also detected processing outside the region of base pairing for both strains (Figure 1A, Supplementary Figure S2B), but it is not clear at this point the origin of these products.

### 5′ end of the OrzO sRNA can solely repress *zorO*

**Figure 3.**
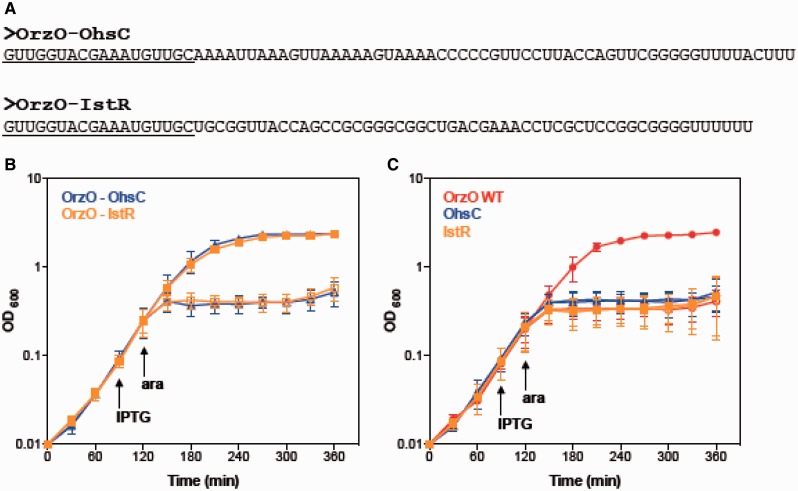
The 5′ end of OrzO is sufficient for inhibition of *zorO*-mediated toxicity. (**A**) Generation of an OrzO-OhsC and OrzO-IstR chimeras. The 5′ ends of the type I antitoxins OhsC and IstR were replaced with the 5′ end of OrzO, the longest region of complementarity to *zorO*. Underlined sequences indicate the OrzO nucleotides. (**B**) Overexpression of both OrzO-OhsC and OrzO-IstR chimeras repress *zorO*. Rescue experiment was performed as outlined in Figure 1B with either OrzO-OhsC (blue triangles) or OrzO-IstR (orange squares) expressed from the P_LlacO-1_ promoter of pBR-plac. Solid symbols indicate the addition of IPTG; open symbols, no IPTG. The averages of three independent cultures ± standard deviations are shown. (**C**) Neither wild type OhsC nor IstR can repress *zorO*-induced toxicity. Rescue experiment was performed with pBR-plac-*orzO* (red circles), pBR-plac-*ohsC* (blue triangles) or pBR-plac-*istR* (orange squares) as outlined in Figure 1B. Solid symbols indicate addition of IPTG; open symbols, no IPTG. Shown are the averages ± standard deviations for three independent cultures.

A previous study has shown that the antitoxin IstR-1 of *E. coli* regulates its toxin target TisB by 5′ end pairing (20). Similarly, the OhsC antitoxin is predicted to base pair to its target ShoB through its 5′ end (18). In *zorO-orzO* pair, the longest stretch of potential base pairing between OrzO and the *zorO* mRNA also occurs in the immediate 5′ end of OrzO. To test if just the 5′ end of OrzO can repress *zorO*, we first constructed two OrzO mutants truncated at the 3′ end. However, we were unable to detect the expression of these truncated sRNAs (data not shown). We hypothesized that the inability to detect these truncated RNAs was owing to their instability; therefore, we generated chimeric sRNAs to the OhsC and IstR-1 sRNAs. The base pairing regions that OhsC and IstR-1 had to their own toxins were replaced with the first 18 nt of the 5′ end of the OrzO sRNA (Figure 3A). Consequently, only the 5′ end of the chimeras can base pair with the *zorO* mRNA. Rescue experiment results showed that although the wild-type OhsC and IstR sRNAs failed to repress *zorO*, both chimeras successfully repressed the expression of *zorO* (Figure 3B and C). This demonstrates that the first 18 nt of OrzO are sufficient for full repression.

### Orz regulation is target specific

**Figure 4.**
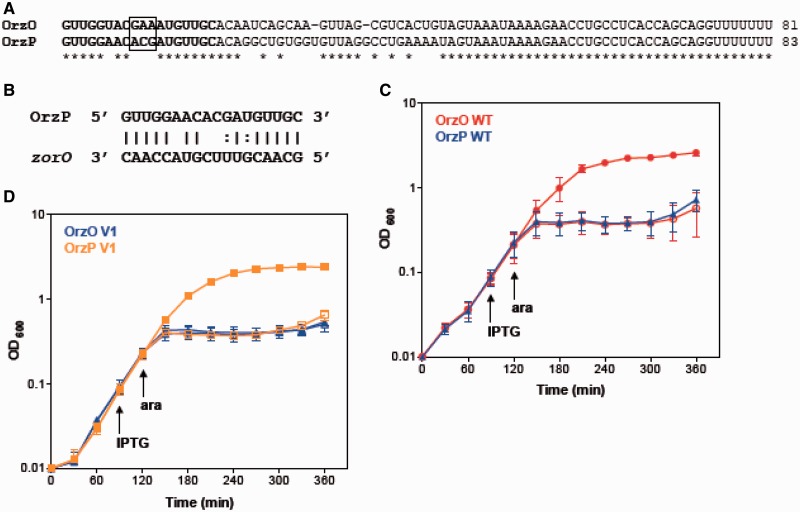
The V1 region is responsible for target discrimination by OrzO. (**A**) Alignment of OrzO and OrzP sRNAs. The V1 region is indicated in the black box; the entire base pairing region is in bold. (**B**) Potential base pairing interactions between OrzP and *zorO* in the predicted binding region. (**C**) OrzO, not OrzP, can repress *zorO*. Rescue experiment was performed as outlined in Figure 1B with either wild-type OrzO (red circles) or OrzP (blue triangles) expressed from the P_LlacO-1_ promoter of pBR-plac. Solid symbols indicate the addition of IPTG; open symbols, no IPTG. Averages ± standard deviations of three independent cultures are shown. (**D**) An OrzP V1 mutant can rescue cells from *zorO* overexpression. Rescue experiment was performed as outlined in Figure 1B using pBR-plac-*orzP* V1 (orange squares), in which its V1 region was mutated to match OrzO, and pBR-plac-*orzO* V1 (blue triangles), in which its V1 was mutated to match OrzP. Solid symbols indicate addition of IPTG; open symbols, no IPTG. Averages ± standard deviations for three independent cultures are shown.

The 5′ end of OrzO contains a continuous stretch of 18 nt that can base pair to the *zorO* mRNA (Figure 1B). The OrzP sRNA, which is a homolog of OrzO, also shares 15 nt complementarity to *zorO* (Figure 4B). Because of the sequence similarities between OrzO and OrzP, it is possible that OrzP is capable of regulating ZorO (Figure 4A).

Rescue experiments were used to determine whether the OrzP sRNA can repress the expression of *zorO*. When OrzO was co-expressed with *zorO*, the strain was able to grow. However, when OrzP was co-expressed with *zorO*, cell stasis occurred (Figure 4C), suggesting that only OrzO, but not OrzP, can repress *zorO*. We also confirmed that OrzP was being expressed at levels equivalent to OrzO by northern analysis (Supplementary Figure S3), which indicates that the failure of OrzP to rescue is not due to expression differences. Thus, repression by the Orz sRNAs is target-specific.

### V1 region determines OrzO specificity for *zorO*

Although OrzO and OrzP share the same predicted base pairing region with *zorO*, only OrzO is able to repress *zorO*. To further investigate what dictates this specificity, we compared the sequences of the two sRNAs (Figure 4A). Despite the great sequence similarity between OrzO and OrzP, there are several differences. In particular, the nucleotide sequences from +9 to +11 compose a variable region of 3 nt between OrzO (GAA) and OrzP (ACG). This variable region (referred to as V1) is the most prominent difference observed within the 5′ regulatory domain of the two sRNAs.

To determine if this V1 region is responsible for specificity in the OrzO regulation of *zorO*, the three nucleotides in the V1 region of OrzO were changed from ‘GAA’ to ‘ACG’, the sequence found in OrzP (Table 1). We then tested the ability of the resulting mutant strain (15 nt of base pairing potential) to rescue cells from *zorO-*induced toxicity. Our results showed that the OrzO V1 mutant completely failed to rescue cells (Figure 4D), suggesting that the V1 region is critical for repression by the OrzO sRNA.

**Table 1.** Summary of the mutations generated and their repressive abilities

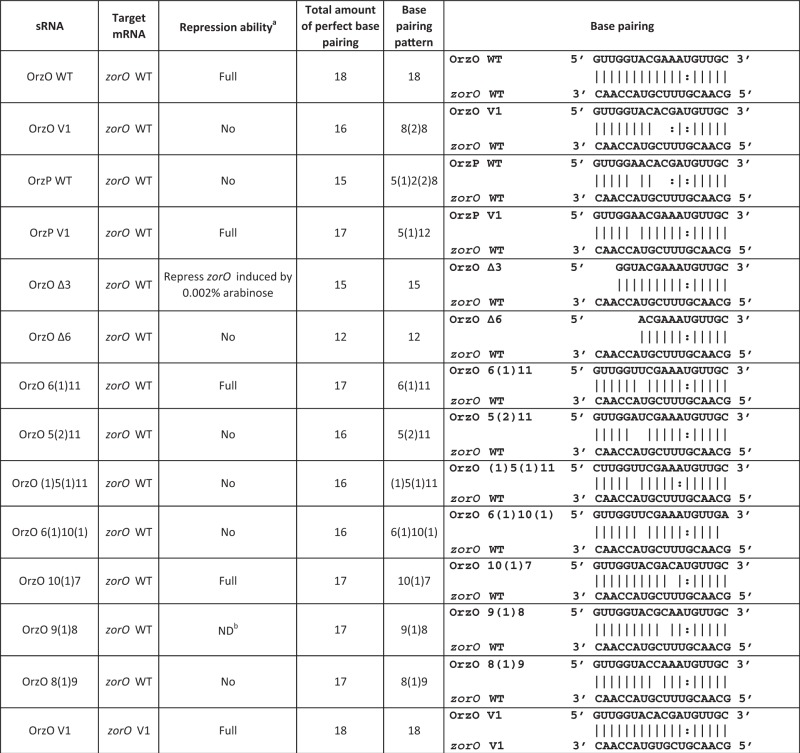

^a^Repression ability when *zorO* induced by 0.2% arabinose unless otherwise indicated.

^b^Despite generation of this mutant independently multiple times, we never obtained consistent results when analyzing its ability to repress *zorO* induced either with 0.2 or 0.002% arabinose.

To confirm these results, we generated compensatory mutations in *zorO* (referred to as *zorO* V1) so as to restore complementarity to the OrzO V1 mutant. Restoring the base pairing allowed the OrzO V1 to fully repress ZorO V1-induced toxicity (Supplementary Figure S4). These data implied that the V1 region is important for OrzO to recognize its target.

Furthermore, we engineered an OrzP V1 mutant, in which its V1 region was changed from ‘ACG’ to ‘GAA’, the sequence found in OrzO. Consequently, the OrzP V1 mutant has increased ability to base pair to *zorO*, as it could perfectly match *zorO* in the V1 region (Table 1). Unlike the OrzP WT, which cannot inhibit *zorO* mRNA expression, the OrzP V1 mutant fully represses *zorO* (Figure 4D). These data, in addition to the observations from the *zorO* WT-OrzO V1 pair and the *zorO* V1-OrzO V1 pair, further support the hypothesis that the V1 region is responsible for the Orz sRNA specificity to its *zor* target.

### The first base of the V1 region is critical for OrzO regulation of *zorO*

Since the V1 region of the 5′ end of OrzO is composed of three nucleotides (GAA), we then asked whether all three nucleotides contribute equally to the ability of OrzO to regulate *zorO*. Each nucleotide of the V1 region was mutated individually and tested for the ability to rescue cells from *zorO* toxicity. The resulting mutants are named OrzO 8(1)9, OrzO 9(1)8 and OrzO 10(1)7. These are named such that the number in the parentheses indicates the number of unpaired nucleotides, while the numbers on the two sides of the parentheses represent the number of the nucleotides within the 5′ end of OrzO that are capable of base pairing with *zorO* (Table 1). For example, in OrzO 8(1)9, the first 8 nt of OrzO can pair to *zorO*, followed by a single unpaired nt, and then 9 nt of pairing.

**Figure 5.**
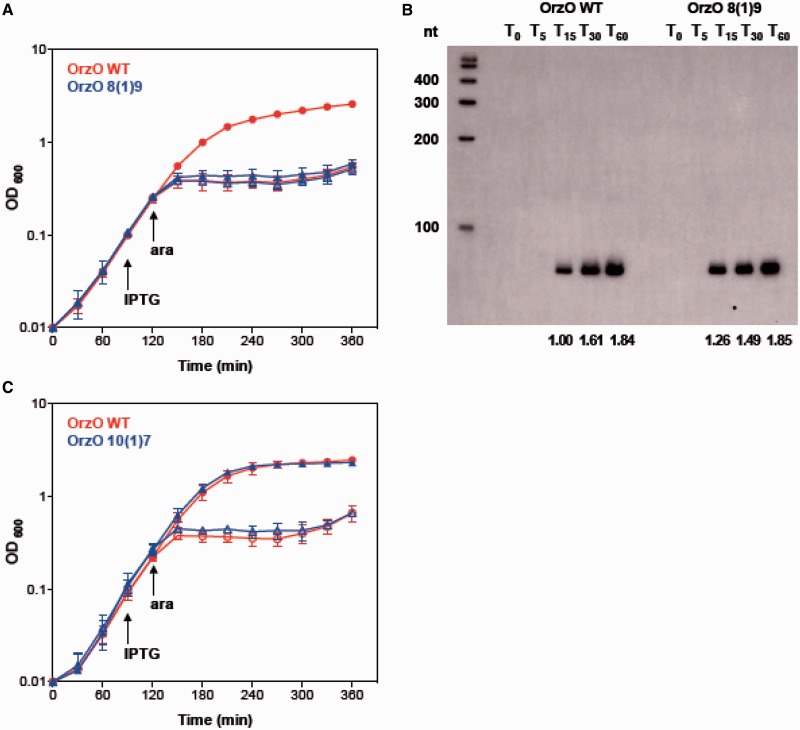
A specific residue within the V1 region of OrzO is necessary for *zorO* repression. (**A**) Mutation of the first nucleotide of the V1 region fails to rescue *E. coli* from ZorO toxicity. Rescue experiment was performed as outlined in Figure 1B except that arabinose was added to a final concentration of 0.002%. Shown are the averages ± standard deviations of pBR-plac-*orzO* (red circles) or pBR-plac-*orzO* 8(1)9 (blue triangles), which has the first nucleotide of the V1 region mutated. Solid symbols indicate addition of IPTG; open symbols, no IPTG. (**B**) Expression of OrzO 8(1)9 is similar to wild type OrzO. Total RNA was isolated from *E. coli* UTK007 overexpressing *zorO* (0.002% arabinose) and either OrzO (1 mM IPTG) or OrzO 8(1)9 (1 mM IPTG) as described in ‘Materials and Methods’ section. Quantification of RNA levels is described in the ‘Materials and Methods’ section, with OrzO WT at T_15_ set to a value of 1. Numbers below the panel indicate the average relative intensities. (**C**) Mutation of the third nucleotide of the V1 region has no impact on the ability of OrzO to repress *zorO* expression. The third nucleotide of the V1 region of OrzO was mutated to generate OrzO 10(1)7. A rescue experiment was performed as outlined in Figure 1B with wild-type OrzO (red circles) or OrzO 10(1)7 (blue triangles) expressed from the P_LlacO-1_ promoter of pBR-plac. Solid symbols indicate addition of IPTG; open symbols, no IPTG. Averages ± standard deviations for three independent cultures are shown.

Rescue experiments were used to assess the repression abilities of the three mutants. Interestingly, the repression ability of the single mutants was not equivalent. The point mutation in the first nucleotide of the V1 region of OrzO completely abolished the repression ability of OrzO sRNA (Figure 5A), as the OrzO 8(1)9 mutant failed to rescue cells even at a low induction level of *zorO* (0.002% arabinose, see below). Northern analysis further indicated that the OrzO 8(1)9 mutant showed levels equivalent to that of the OrzO wild type (WT) at T_15_ and T_60_, suggesting that the failure of the OrzO 8(1)9 mutant to rescue is due to mutating the first nucleotide of the V1 region (Figure 5B, Supplementary Figure S7A). Mutation of the third nucleotide of the V1 region did not affect the repression ability of OrzO, as the OrzO 10(1)7 mutant still fully repressed *zorO* (Figure 5C).

The OrzO 9(1)8 mutant (second nucleotide of the V1 mutated) was highly variable in its ability to repress *zorO* (Supplementary Figure S6A), despite generation of the mutant independently multiple times. To clarify whether this may be due to expression problems, we performed a more in-depth expression comparison to wild type OrzO. We first examined the basal level of OrzO induction required to repress *zorO*, and determined that this occurs at 0.1 mM IPTG induction (Supplementary Figure S5A–C). We then compared expression of OrzO 9(1)8 when induced at 1 mM IPTG to wild type OrzO expressed at 0.1 mM IPTG (Supplementary Figure S6B and C). Indeed, expression of OrzO 9(1)8 is higher than the basal levels of wild-type OrzO that is needed for *zorO* repression. This suggests that the attenuated repression ability observed in OrzO 9(1)8 is likely due to the nucleotide change rather than the expression difference. Regardless, our results imply that the first nucleotide of the V1 region is most critical for the OrzO sRNA to regulate *zorO*.

### 15 nt of continuous base pairing interactions are sufficient for OrzO regulation

We have shown that the 5′ base pairing domain of OrzO is critical for regulating *zorO*, and the V1 region dictates the correct recognition of the cognate *zor* targets. However, it is not clear whether all nucleotides within the 5′ end of OrzO besides the V1 region are needed for repression of *zorO*. To better refine the requirements for successful base pairing, we generated two deletion constructs in which the first 3 and 6 nt were deleted from the 5′ end of OrzO while leaving the V1 intact. The OrzO Δ3 and Δ6 share a continuous stretch of 15 and 12 nt complementarity with *zorO* mRNA, respectively (Table 1).

**Figure 6.**
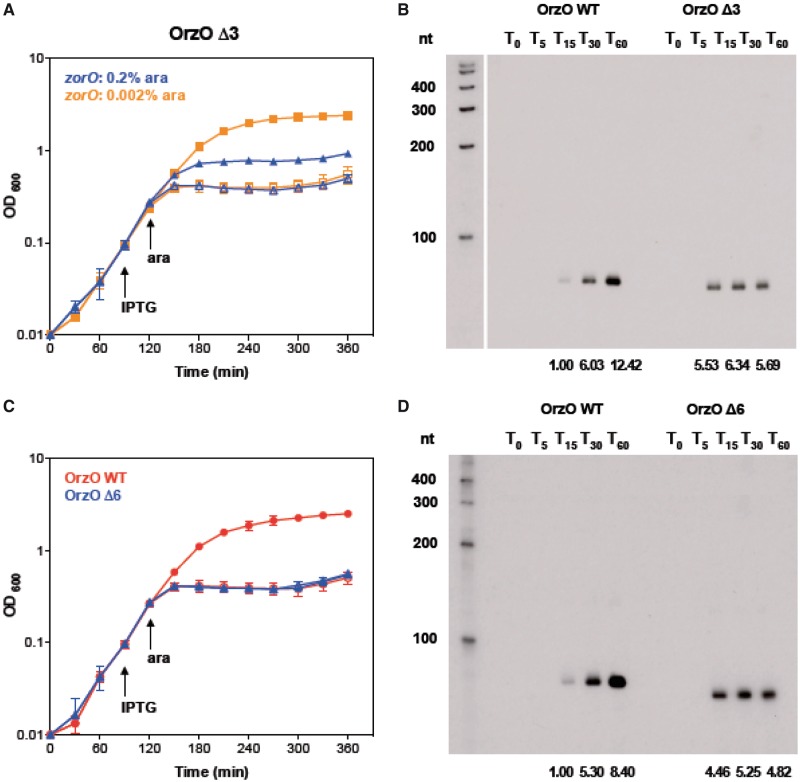
Only 15 nt of continuous base pairing are required for OrzO inhibition of *zorO*. (**A**) OrzO Δ3 prevents *zorO*-induced toxicity at low levels of arabinose. Rescue experiment was performed as outlined in Figure 1B with OrzO Δ3 expressed from the P_LlacO-1_ promoter of pBR-plac. Note that *zorO* was induced by either 0.2 (blue triangles) or 0.002% (orange squares). Solid symbols indicate addition of IPTG; open symbols, no IPTG. Averages ± standard deviations for three independent cultures are shown. (**B**) Comparison of RNA levels between wild-type OrzO induced by 0.1 mM IPTG and OrzO Δ3 induced by 1 mM IPTG. Total RNA was isolated from *E. coli* UTK007 overexpressing OrzO or OrzO Δ3 from pBR-plac as described in ‘Materials and Methods’ section. Average intensities are shown as described in Figure 5B and in the ‘Materials and Methods’ section. **(C)** OrzO Δ6 fails to repress *zorO*-induced toxicity even at low levels of arabinose induction. Rescue experiment was performed as outlined in Figure 5A using either pBR-plac-*orzO* (red circles) or pBR-plac-*orzO* Δ6 (blue triangles) as indicated. Note that the arabinose concentration used was 0.002%. Solid symbols indicate addition of IPTG; open symbols, no IPTG. Averages ± standard deviations for three independent cultures are shown. **(D)** Comparison of RNA levels between the wild-type OrzO induced by 0.1 mM IPTG and the OrzO Δ6 mutant induced by 1 mM IPTG. Total RNA was isolated from *E. coli* UTK007 overexpressing OrzO or OrzO Δ6 from pBR-plac as described in ‘Materials and Methods’ section. Average intensities are shown as described in Figure 5B and in the ‘Materials and Methods’ section.

Rescue experiments were used to assess the abilities of these two mutants to repress *zorO*. Neither the OrzO Δ3 nor Δ6 completely repressed *zorO* when we used 0.2% arabinose to induce expression of the toxin (Figure 6A, data not shown). We also inspected the levels of OrzO Δ3 and Δ6 and observed that their levels were decreased as compared with the level of full-length OrzO when all were induced with 1 mM IPTG (data not shown).

On closer examination of the rescue experiment using OrzO Δ3, we noticed a ‘transient’ rescue or a delay in growth stasis compared with expression of *zorO* alone when induced with 0.2% arabinose. When we reduced the arabinose concentration to 0.002%, we noted that overproduction of ZorO still caused cell growth stasis, in a pattern identical to the 0.2% arabinose induction group (Supplementary Figure S5D and E). In addition, co-expression of OrzO Δ3 relieved the toxicity caused by induction of *zorO* at 0.002%, suggesting that the failure of this mutant to rescue at higher concentration of arabinose was likely due to the reduced levels of this sRNA mutant (Figure 6A). However, OrzO Δ6 still could not repress toxicity when ZorO was induced by 0.002% arabinose (Figure 6C).

We observed that the RNA levels of OrzO Δ3 and Δ6 (induced by 1 mM IPTG) were higher than the basal levels of wild-type OrzO (induced by 0.1 mM IPTG) at T_15_ (Figures 6B, D, Supplementary Figure S7B and C). The levels of the wild type sRNA continued to increase over time (T_30_ and T_60_), whereas the levels of the truncated sRNAs remained relatively steady. Even though OrzO Δ3 did not accumulate to a level as high as the wild-type sRNA, it was capable of repressing *zorO*. OrzO Δ6, however, could not even transiently rescue cells, despite being induced initially to levels higher than wild type OrzO (Figure 6D, Supplementary Figure S7C). Combined, these data imply that 15 nt, but not 12 nt, of continuous base pairing is sufficient for the OrzO sRNA to repress *zorO.*

### A minimum of 17 nt of discontinuous base pairing interactions is required for OrzO repression of *zorO*

As shown above, 15 nt of continuous complementarity to *zorO* is sufficient for repression. Our OrzP V1 mutant can also repress *zorO*, but it does not have 15 nt of continuous base pairing (Table 1). Instead, this mutant has 17 nt of discontinuous pairing. This suggests that the amount of complementary base pairs required for discontinuous base pairing interactions may be different from that for continuous base pairing interactions.

**Figure 7.**
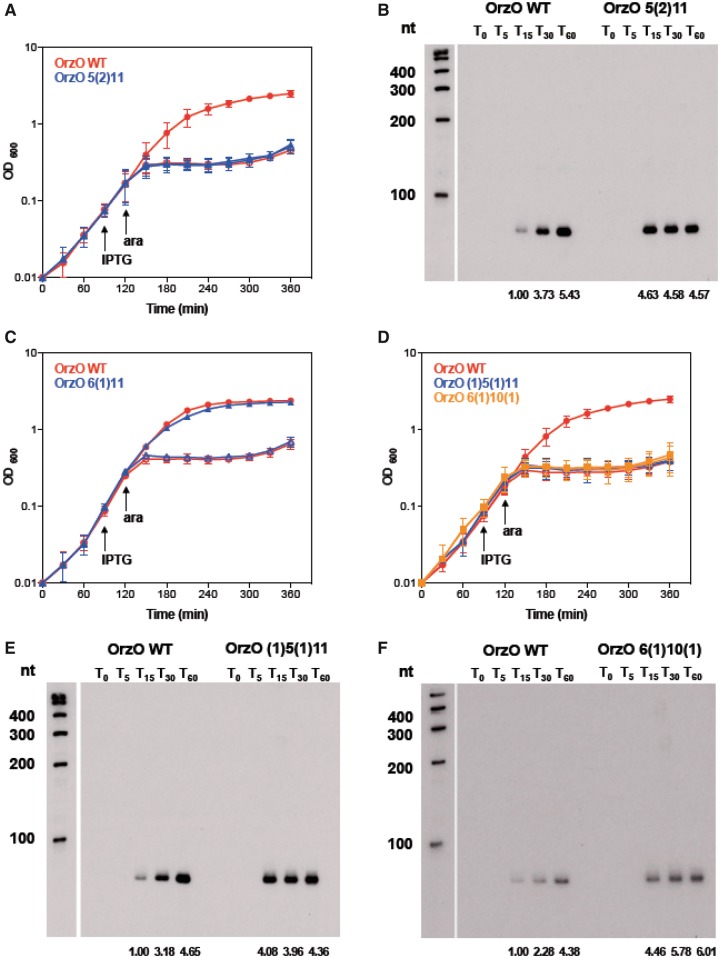
Repression of *zorO* requires 17 nt of discontinuous base pairing. (**A**) OrzO 5(2)11 fails to inhibit ZorO toxicity. Rescue experiment was performed as outlined in Figure 5A with either wild-type OrzO (red circles) or OrzO 5(2)11 (blue triangles) expressed from the P_LlacO-1_ promoter of pBR-plac. Solid symbols indicate addition of IPTG; open symbols, no IPTG. Averages ± standard deviations for three independent cultures are shown. (**B**) Comparison of RNA levels between the wild type OrzO induced by 0.1 mM IPTG and the OrzO 5(2)11 mutant induced by 1 mM IPTG. Average intensities are shown as described in Figure 5B and in the ‘Materials and Methods’ section. (**C**) OrzO 6(1)11 can repress *zorO*. Rescue experiment was performed as outlined in Figure 1B with either wild-type OrzO (red circles) or OrzO 6(1)11 (blue triangles) expressed from the P_LlacO-1_ promoter of pBR-plac. Solid symbols indicate addition of IPTG; open symbols, no IPTG. Averages ± standard deviations for three independent cultures are shown. (**D**) Neither OrzO 6(1)10(1) nor OrzO (1)5(1)11 can prevent *zorO*-induced toxicity. Rescue experiment was performed as outlined in Figure 5A with pBR-plac-*orzO* (red circles), pBR-plac-*orzO* (1)5(1)11 (blue triangles) or pBR-plac-*orzO* 6(1)10(1) (orange squares). Note that IPTG was added to 1 mM final concentration, and arabinose was added to 0.002% final concentration. Solid symbols indicate addition of IPTG; open symbols, no IPTG. Averages ± standard deviations for three independent cultures are shown. (**E**) Comparison of RNA levels between the wild-type OrzO induced by 0.1 mM IPTG and the OrzO (1)5(1)11 mutant induced by 1 mM IPTG. Average intensities are shown as described in Figure 5B and in the ‘Materials and Methods’ section. **(F)** Comparison of RNA levels between the wild-type OrzO induced by 0.1 mM IPTG and the OrzO 6(1)10(1) mutant induced by 1 mM IPTG. Average intensities are shown as described in Figure 5B and in the ‘Materials and Methods’ section.

Given that the OrzP V1 [pairing: 5(1)12] can rescue cells from *zorO*-induced toxicity, we designed series of mutants with decreased base pairing potential as compared with OrzP V1 (Table 1). We constructed OrzO 5(2)11 with 16 nt of base pairing, which is composed of a 5-nt stretch of complementarity, followed by a 2-nt gap and then another 11-nt stretch of complementarity. The OrzO 5(2)11 mutant could not repress ZorO toxicity when the mRNA was induced with either 0.2 or 0.002% arabinose (Figure 7A and data not shown). We also confirmed that the OrzO 5(2)11 mutant (induced by 1 mM IPTG) was being expressed at levels higher than the basal levels of OrzO WT (induced by 0.1 mM IPTG) at T_15_ and T_30_ (Figure 7B, Supplementary Figure S7D), suggesting that the failure of OrzO 5(2)11 to rescue is likely not due to insufficient RNA levels, but rather base pairing differences.

Our OrzO 5(2)11 possesses 16 nt of pairing to *zorO*, whereas our OrzP V1 possesses 17 nt of pairing [5(1)12]. It maybe that a minimum of 17 nt of discontinuous base pairing is required for repression. To explore this further, we generated several more constructs. The OrzO 6(1)11 mutant has 17 nt of pairing, and indeed it can fully repress *zorO* (Figure 7C). We then mutated the last nucleotide of pairing of this construct, generating the OrzO 6(1)10(1) mutant, which possess 16 nt of pairing. We also generated the OrzO (1)5(1)11 mutant, in which the first nucleotide of pairing was mutated, and in total, possesses only 16 nt of pairing. When either mutant was used in a rescue experiment, they were unable to repress toxicity, even if *zorO* was induced with 0.002% arabinose (Figure 7D). Furthermore, northern analysis indicated that these mutants (induced by 1 mM IPTG) were expressed at levels equivalent to or even higher than OrzO WT induced by 0.1 mM IPTG (Figure 7E, F, and Supplementary Figure S7E, F). Thus, the failure to rescue is likely due to insufficient pairing and not due to insufficient production of the antitoxin.

## DISCUSSION

In this study, we show that the *zorO-orzO* gene pair of *E. coli* O157:H7 is a true type I toxin–antitoxin locus. More specifically, our data are consistent with the properties of an unconventional type I system, as the OrzO antitoxin is encoded divergently from *zorO* and has limited base pairing potential with it. Overproduction of ZorO is toxic; however, co-expression of OrzO represses this toxicity and rescues the cells. We discovered that the 5′ end of OrzO can solely repress *zorO*-induced toxicity, and the V1 region dictates the correct recognition of *zorO*. Furthermore, the first base of the V1 region is critical for the OrzO sRNA to regulate the expression of *zorO* mRNA. Although there is 18-nt complementarity with *zorO* at the 5′ end of OrzO, not all base pairing interactions are needed for repression. For continuous complementarity, 15 nt of perfect base pairing is sufficient to function, while for discontinuous complementarity, a minimum of 17 nt of perfect base pairing is required to maintain the repression ability if a single non-pairing nucleotide is present within the base pairing region.

### Role of RNase III in repression of *zorO* expression

Ribonuclease III (RNase III), encoded by the *rnc* gene, cleaves double-stranded RNA, and has been linked to processing of several type I toxin–antitoxin pairs found in *E. coli* and *B. subtilis* (16,20,34). While RNase III is not essential for growth in *E. coli*, deletion of the gene in *B. subtilis* is lethal. Work by Durand *et al.* has shown that the essentiality of RNase III in *B. subtilis* is because it cleaves the type I toxins *txpA* and *yonT* (36). Interestingly, while the RNA duplex of another type I toxin–antitoxin locus in *B. subtilis*, BsrG/SR4, is cleaved by RNAse III, repression of the toxin does not require this ribonuclease (16). Thus, while RNase III plays a role in the processing of several described type I pairs in both *E. coli* and *B. subtilis*, its essentiality for repression can be variable between the different systems.

RNase III is also involved in the processing of other RNA duplexes that are not type I toxin–antitoxin systems. These include the interaction of the mRNA *gadX-gadW* with the sRNA GadY and the interaction of the mRNA *cII-O* with the OOP RNA of λ (35,38,39). For the *zorO-orzO* pair, deletion of RNase III led to accumulation of the full-length *zorO* mRNA, and in this strain background, *zorO*-induced toxicity could not be repressed by co-expression of OrzO. Yet, we still could detect OrzO-dependent processing of *zorO* in a *Δrnc* strain. Similar observations were seen with *gadX-gadW* and *cII-O*; cleavage still occurred without RNase III, although the processing sites were slightly shifted (35,38,39). We observed a similar shift in the processing of *zorO* in a *Δrnc* strain (Figure 1A, Supplementary Figures S2B). There are other ribonucleases within *E. coli* that may be contributing to this processing [reviewed in (40)]. For example, RNaseE is critical for the function of many Hfq-dependent base pairing RNAs [reviewed in (41)]. Further analysis is needed to determine what other enzymes may be contributing to processing of *zorO*-OrzO; however, based on the in-depth analysis of the *gadX-gadW* processing by GadY, it is possible that an uncharacterized double-stranded ribonculease may exist (35).

We were surprised to note that the levels of OrzO were reduced in a *Δrnc* strain; we have observed that the chromosomal expression levels of OrzO within an *E. coli* O157:H7 deleted for *rnc* are also decreased in comparison with a wild-type strain (Won and Fozo, unpublished observations). We had anticipated that the levels of OrzO would either be unaffected by the loss of RNase III or perhaps higher, as there would be decreased cleavage of the *zorO*-OrzO RNA duplex. It maybe that RNase III cleaves OrzO directly, and that this cleavage impacts its stability and/or activity. However, we have been unable to detect alternative forms of OrzO, and cannot conclude that this is occurring.

### Regulation of ZorO by the 5′ end of OrzO

To determine whether the 5′ end of OrzO was sufficient for regulating *zorO*, we generated two constructs expressing either the first 42 or 34 nt of OrzO. These truncated sRNAs did not rescue, nor were we ever able to detect their overexpression (data not shown). However, our chimeric sRNAs, OrzO-OhsC (75 nt) and OrzO-IstR (72 nt) were stable and able to rescue *E. coli* from ZorO toxicity. Thus, although the 5′ end of OrzO is sufficient for repression, additional RNA sequence at the 3′ end may be needed for stability. Many type I antitoxins are predicted to have extensive secondary structure (7). The reason for such extensive structure among the type I antitoxins has not been thoroughly examined, but our data suggest that it may be required to prevent quick degradation of the sRNA.

Our data show that the OrzO sRNA represses *zorO* expression through base pairing interactions between the 5′ end of OrzO and the 5′ UTR of *zorO*. The binding region in the 5′ end appears to be conserved across many sRNAs. For example, a previous study that examined 18 well-characterized *E. coli/Salmonella* Hfq-dependent sRNAs found that more than one-third used their 5′ ends to repress their targets (42). In addition, the Hfq-independent sRNA IstR-1 also acts by 5′ end pairing [reviewed in (19)]. Similar interactions have been predicted to occur with the *shoB-ohsC* pair, in which the 5′ end of OhsC shares 19 nt of complementarity to the 5′ UTR of *shoB* [(18) and reviewed in (19)]. Although the reason why so many sRNAs function through 5′ pairing remains unclear, it has been proposed that similar to eukaryotic microRNAs, some bacterial sRNAs may have a 5′ conserved ‘seed’ region that facilitates the selection for correct mRNA targets (42). Thus, the OrzO sRNA likely uses the first 18 nt of its 5′ end as the ‘seed’ region. Further sequence analysis studies of a larger group of sRNAs and their targets will be helpful to evaluate whether the existence of the ‘seed’ region can be applied as a general principle in the prediction of bacterial 5′ pairing sRNAs and their target mRNAs.

### Recognition of *zorO* by the V1 region of OrzO

We have shown specifically that the V1 region of OrzO plays a crucial role in recognizing its target. Replacing the V1 region of OrzO with that of OrzP abrogates the ability of the sRNA to repress the *zorO* mRNA, whereas replacing the V1 region of OrzP with that of OrzO allows the mutated OrzP to repress *zorO*.

**Figure 8.**
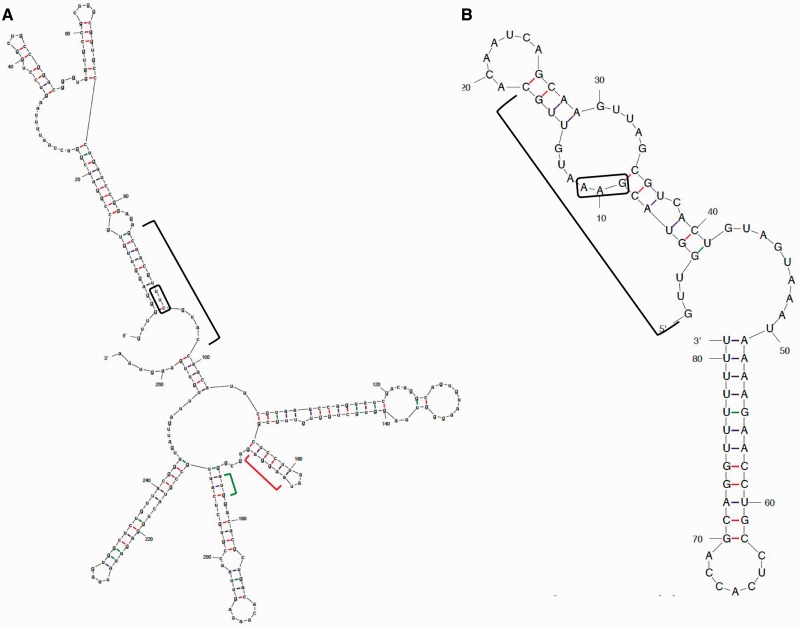
Predicted secondary structures of *zorO* mRNA and OrzO indicate the V1 regions are mostly single-stranded. (**A**) Putative RNA structure of *zorO* mRNA generated by M-fold (http://http://mfold.rna.albany.edu/?q=mfold/RNA-Folding-Form) (43). The black bracket indicates the region of complementarity to OrzO; red brackets, the putative ribosome binding site; green bracket, the start codon. The black box is the V1 region binding site. (**B**) Putative RNA structure of OrzO sRNA generated by M-fold (http://mfold.rna.albany.edu/?q=mfold/RNA-Folding-Form) (43). The sequence indicated by the black bracket is the region of complementarity to the *zorO* mRNA; the black box is the V1 region.

When inspecting the predicted structures of OrzO and *zorO* (Figure 8A and B), we find that both the V1 region of OrzO and the V1 target site in *zorO* are located in single-stranded structures. The relatively relaxed property of these single-stranded structures may allow for enhanced access of the single-stranded nucleotides to pair with their targets compared with those in a stem structure. Thus, it is likely that the pairing between the V1 region of OrzO sRNA and the corresponding site of *zorO* mRNA occurs before that of other complementary nucleotide regions.

In addition, when we mutated the bases of the V1 region, we noted that mutation of the first nucleotide of the V1 region caused a complete loss of the repression of *zorO* without affecting the levels of the sRNA. However, other single base mutants constructed in this study all successfully repress *zorO* (Table 1). These results suggested that as compared with other nucleotides, pairing via the first nucleotide of the V1 region is most critical for the OrzO sRNA to regulate *zorO* mRNA expression.

These observations suggest that OrzO and *zorO* may interact through a ‘kissing complex’. Generally in a ‘kissing complex’, the binding regions of the sRNA and the mRNA are located in loop structures (single-stranded). The initial base pairing occurs at the short exposed regions; thus, mutations of nucleotides involved the primary ‘kissing’ region impact the repression outcome, as exemplified by the RNAII-RNAI pair in ColE1-derived plasmid and the CopA-CopT pair in R1 plasmid (44,45). Once the initial pairing is established, the kissing complex can extend and propagate the formation of double-stranded RNA in the rest of the sequence. Similarly, this has been reported for the interaction of the *ibs*-Sib type I toxin–antitoxin pair in *E. coli*; however, in this system, there are two recognition sites, in *zorO-orzO*, there appears to be only one recognition domain (46).

While these previously described systems seem to corroborate the results of this study, structure-probing and kinetic experiments need to be performed to validate the predictions and to define the order of occurrence of base pairing in different regions.

### Multiple factors contribute to the repression ability of Orz

Antitoxin sRNAs function by base pairing to their target mRNAs. To successfully repress the toxin mRNA, an adequate amount of functional antitoxin sRNA is required. Otherwise, even if the sRNA is capable of repressing its mRNA target, insufficient RNA levels will result in partial or complete loss of repression.

We observed this directly with our OrzO Δ3 mutant, the expression levels of which were lowered when compared with full-length OrzO. Because all of our Orz constructs are under the control of the same promoter (IPTG-inducible P_Llac-O1_), the decreased levels of the OrzO Δ3 are likely due to accelerated degradation. When performing the rescue experiment using the OrzO Δ3 mutant, an initial repression of *zorO* was observed when we induced the toxin with 0.2% arabinose, but this repression was not long lasting (Figure 6A). Because the antitoxin sRNA is induced 30 min before that of the toxin mRNA, it is likely that enough OrzO Δ3 sRNA had accumulated to initially repress *zorO*; however, as the ‘pre-made’ OrzO Δ3 was consumed through base pairing with *zorO*, the newly produced OrzO Δ3 was not sustained at a high enough level to maintain *zorO* repression. When the inducer concentration was decreased from 0.2 to 0.002% to reduce *zorO* levels, the OrzO Δ3 mutant was able to repress *zorO*, implying that the levels of the sRNA were now sufficient for repression.

It is also likely that the structure, and thus, specific nucleotide sequence, of OrzO contributes as well to its functional ability. For example, several of our mutants showed reduced expression levels at 1 mM IPTG when compared with the wild type at the same IPTG induction. As stated, all of our OrzO constructs were controlled by the same inducible promoter, so transcription should not be affected. Thus, it is more likely that these constructs are more unstable than wild-type OrzO. As shown in Figure 8, much of the 5′ end of OrzO (region of base pairing) is predicted to be in a stem structure. Disruption of this long stem, even by a single nucleotide, may impact overall stability of the sRNA. Further analysis of the effect of these mutations on the structure and degradation of OrzO is needed to resolve this.

Aside from RNA levels, the base pairing potential of the Orz sRNA also contributes to its ability to repress *zorO*. Our data show that 15 nt of continuous complementarity at 5′ end of OrzO are sufficient for the repression of *zorO* mRNA, as represented by the OrzO Δ3 mutant. However, unlike continuous base pairing, in which the pairing region of the sRNA perfectly matches the target sequence, unpaired nucleotides would interrupt the formation of the sRNA-mRNA duplex, affecting repression of the mRNA. Thus, it is likely that more base pairing interactions are needed to compensate for the instability of the RNA duplex caused by the unpaired base(s). The OrzO 6(1)10(1) and 6(1)11 mutants support this theory. Both RNAs were produced at levels similar to wild-type OrzO, but only Orzo 6(1)11 was able to successfully repress ZorO toxicity. The main difference between OrzO 6(1)10(1) and OrzO 6(1)11 comes from the total number of base pairing interactions, 16 versus 17, respectively. Additionally, the OrzP V1 mutant [pairing: 5(1)12] also successfully represses *zorO*. Combined, these data suggest that the total amount of pairing impacts the repression ability, rather than specific positioning of nucleotides (outside the V1 region).

### How does binding to the 5′ UTR repress *zorO* toxicity?

The region of complementarity in the *zorO* mRNA is located in its long 5′ UTR, ∼73–91 nt upstream of the start codon. How could binding to this region prevent *zorO* expression? It is important to note that regulation of the type I toxins Hok and TisB by their respective antitoxins occurs through pairing far upstream of the start codons (47,48). Translation of *hok* requires the translation of a small peptide (encoded by *mok*) found within the 5′ UTR of the *hok* mRNA. The antitoxin, Sok, is complementary to *mok*, and hence binding of the sRNA prevents translation of *mok*, and consequently, *hok* (48). For *tisB*, its ribosome binding site is inaccessible owing to its secondary structure. However, within its 5′ UTR is a standby ribosome binding site; here the ribosome can bind, and as the structure breathes, move to the true ribosome binding site for translating *tisB*. The antitoxin, IstR-1, has sequence complementarity that overlaps the stand-by site; thus, formation of a *tisB*-IstR-1 RNA duplex would prevent ribosome binding (47).

The *zorO* mRNA has 5′ UTR that is ∼180 nt in length (Figure 1A); within this UTR are several potential small open reading frames and ribosome binding sites. It is possible that *zorO* translation is mediated in a manner similar to either *hok* or *tisB*, and that binding by OrzO may block either translation of an internal peptide or binding of the ribosome to a standby site. Ongoing experiments will help elucidate how binding of the antitoxin so far upstream from the ATG start site successfully represses *zorO* expression.

### Role of the *zor-orz* locus?

A major question regarding many type I toxin-antitoxin loci is what is their true biological function? This is particularly true for the chromosomally encoded loci. Work has implicated the role of the type I toxin TisB in halting cell division during the SOS response, allowing the cell time to repair damaged DNA, as well as playing a role in persister cell formation (49,50). Several of the type I loci described in *B. subtilis* exist on prophages [reviewed in (51)]. In particular, *txpA-ratA* is located on the *skin* element, which is excised during sporulation (17). It has been suggested that TxpA helps maintain the *skin* element within *B. subtilis*. For the BsrG/SR4 locus, data have shown that degradation of the *bsrG* toxin mRNA is accelerated under high temperatures (16). How this may influence the function of the toxin has not yet been elucidated.

In the case of the *zor-orz* locus, it is not found beyond *E. coli* and *Shigella* species, and is absent from laboratory strains of *E. coli* like MG1655 (14). This implies that the locus may have been lost on domestication of *E. coli* and perhaps the *zor-orz* locus is needed for growth in the normal habitat *of E. coli*, the intestine. Furthermore, previous work showed that there are differences in the chromosomal expression of *zorO* and *orzO* during growth with glucose as the sole carbon source (14). We have confirmed that the endogenous expression of the sRNA and the mRNA is sensitive to carbon source, implying a possible role for the locus in cellular metabolism (Wen, Fozo, unpublished observations). Thus, the *zor-orz* locus may play a role in controlling cellular growth in response to changing nutrient conditions in the native environment. Current work examining the effects of deleting the locus on total cellular metabolism and gene expression are ongoing to decipher how these genes may impact overall cellular growth.

Overall, our data indicate that the 5′ end of OrzO is sufficient for repressing *zorO*; that the V1 region dictates Orz sRNA specificity for its target; and that there appears to be a minimum length required for base pairing. Further investigations are aimed at understanding the precise regulatory mechanisms of this untraditional type I toxin–antitoxin system to better our understanding of the intricacies of RNA regulation.

## SUPLLEMENTARY DATA

Supplementary Data are available at NAR Online.

## FUNDING

Funding for this work was provided by start-up funds from the University of Tennessee. Funding for open access charge: University of Tennessee start-up funds.

*Conflict of interest statement*. None declared.

## Supplementary Material

Supplementary Data
