# Supplementary material for: The ZorO-OrzO type I toxin–antitoxin locus: repression by the OrzO antitoxin
Source: Nucleic Acids Res. 2013 Nov 6;42(3):1930–46. doi: 10.1093/nar/gkt1018 (PMC3919570; doi:10.1093/nar/gkt1018)
Supplement: Supplementary Data [file supp_42_3_1930__index.html]

The ZorO-OrzO type I toxin–antitoxin locus: repression by the OrzO antitoxin — The ZorO-OrzO type I toxin–antitoxin locus: repression by the OrzO antitoxin — Supplementary Data 

# The ZorO-OrzO type I toxin–antitoxin locus: repression by the OrzO antitoxin

## Supplementary Data

files

**Files in this Data Supplement:**

- Supplementary Data - pdf file
