# Supplementary material for: The ZorO-OrzO type I toxin–antitoxin locus: repression by the OrzO antitoxin
Source: Nucleic Acids Res. 2013 Nov 6;42(3):1930–46. doi: 10.1093/nar/gkt1018 (PMC3919570; doi:10.1093/nar/gkt1018)
Supplement: Supplementary Data [file supp_gkt1018_nar-01581-v-2013-File002.pdf]

Supplementary Table S1. Strains used in this study.

Name	Relevant genotype or description	Source
Escherichia coli EDL933	Wildtype EHEC, O157:H7	D. Friedman
DJ480	MG1655 *ΔlacX74* (wild type strain)	D. Jin, (20)
UTK007	DJ480 PCP18-*araE*	This study
UTK008	UTK007 Δ*hfq::kan*	This study
UTK011	UTK007 Δ*rnc::kan*	This study
Plasmids		
pKD4	Km^R	(24)
pBR-pLac	Amp^R; P_{LacO} promoter	(20)
pEF21	Cm^R; P_{BAD} promoter	(17)
pBR-pLac-*orzO*	AmpR	This study
pEF21-*zorO*	Cm^R	This study
pBR-pLac-*orzO-ohsC*	Amp^R	This study
pBR-pLac-*orzO-istR*	Amp^R	This study
pBR-pLac-*orzP*	Amp^R	This study
pBR-pLac-*orzO* V1	Amp^R	This study
pEF21-*zorO* V1	Cm^R	This study
pBR-pLac-*orzP* V1	Amp^R	This study
pBR-pLac-*orzO* 8(1)9	Amp^R	This study
pBR-pLac-*orzO* 10(1)7	Amp^R	This study
pBR-pLac-*orzO* 9(1)8	Amp^R	This study
pBR-pLac-*orzO*Δ3	Amp^R	This study
pBR-pLac-*orzO*Δ6	Amp^R	This study
pBR-pLac-*orzO* 5(2)11	Amp^R	This study
pBR-pLac-*orzO* 6(1)11	Amp^R	This study
pBR-pLac-*orzO* 6(1)10(1)	Amp^R	This study
pBR-pLac-*orzO* 1(5)1(11)	Amp^R	This study

Supplementary Table S2. Oligonucleotides used in this study

Name	Sequence (5'-3', restriction sites underlined; nucleotides altered via site directed mutagenesis in red)	Use
EF506	CCTGCTGGTGAGGCAGGTTCTTTTATTT	OrzO Northern analysis
EF511	GAGCACGGTTAACTTTTGTGTCAGCGTGT	*zorO* Primer extension analysis
EF517	CTACAGTGACGCTAACTTGCTGATT	OrzO Northern analysis
EF524	GTTGGTACGAAACGTTGCTCTCCG	*zorO* Northern analysis
EF769	CAGAATCGAAAGGTTCAAAGTACAAATAAGCATATAAGGAAAAGAGAGAATGATTGAACAAGATGGATTG	UTK008 PCR (Δ*hfq*)
EF770	CGCAGGATCGCTGGCTCCCCGTGTAAAAAAAACAGCCCGAAACCTTAGAAGAACTCGTCAAGAAG	UTK008 PCR (Δ*hfq*)
EF771	GTCTGTTTCGTGTGCTGAATTGTTGACGCATTTATTTATTGGTATCGCATGATTGAACAAGATGGA	UTK011 PCR (Δ*rnc*)
EF772	GATGGCAATAAATCCGCAGTAACTTTTATCGATGCTCATTCCAGCTCCAGTCAGAAGAACTCGTCAAGAAG	UTK011 PCR (Δ*rnc*)
EF537	GTGGATATGGTTATAATGACGTCGTTGTTACGAA	pBR-pLac-*orzO* PCR
EF538	GATGTGGAATTCTATGGTGGGCTATTGGC	pBR-pLac-*orzO* PCR; pBR-pLac-*orzO-ohsC* PCR; pBR-pLac-*orzO-istR* PCR; pBR-pLac-*orzO*Δ3 PCR, pBR-pLac-*orzO*Δ6 PCR
EF531	GAGATTTATAATCCTGCAGGTTGGGACGTTGC	pEF21-*zorO* PCR
EF532	GAGTTAAAGCTTGAATTAATAAAAACAGTAATC	pEF21-*zorO* PCR
EF78	GACACGGAAATGTTGAATAC	pBR-pLac-orzO-ohsC PCR; pBR-pLac-orzO-istR PCR
EF790	CTTTTTAACTTTAATTTTGCAACATTTCGTACCAACGACGTC	pBR-pLac-*orzO-ohsC* PCR
EF791	GAAATGTTGCAAAATTAAAGTTAAAAAGTAAAACCCCCGTTCC	pBR-pLac-*orzO-ohsC* PCR
EF792	GCCACGGTAA GAATTC AAAGTTAAAAATAATACCG	pBR-pLac-*orzO-ohsC* PCR
EF793	GCGGCTGGTAACCGCAGCAACATTTCGTACCAACGACGTC	pBR-pLac-*orzO-istR* PCR
EF794	CGAAATGTTGCTGCGGTTACCAGCCGCGGGCGGCTGACG	pBR-pLac-*orzO-istR* PCR
EF795	CTGTACTGCAGAATTCAAAAAACCCCGCCGGAGCG	pBR-pLac-*orzO-istR* PCR
EF541	CCAGCCGTGGTTATAATGACGTCGTTGGAACACG	pBR-pLac-*orzP* PCR
EF542	GTTGTTGAATTCAAAAATATCTGGG	pBR-pLac-*orzP* PCR
EF700	CTGACGTCGTTGGTAC**ACG**ATGTTGCACAATCAGC	pBR-pLac-*orzO* V1 PCR
EF701	GCTGATTGTGCAACAT**CGT**GTACCAACGACGTCAG	pBR-pLac-*orzO* V1 PCR
EF725	GATCCGGAGAGCAACGT**CGT**GTACCAACATACGTAAAC	pEF21-*zorO* V1 PCR
EF726	GTTTACGTATGTTGGTAC**ACG**ACGTTGCTCTCCGGATC	pEF21-*zorO* V1 PCR
EF702	CTGACGTCGTTGGAAC**GAA**ATGTTGCACAGGCTGTG	pBR-pLac-*orzP* V1 PCR
EF703	CACAGCCTGTGCAACAT**TTC**GTTCCAACGACGTCAG	pBR-pLac-*orzP* V1 PCR
EF998	CTGACGTCGTTGGTAC**C**AAATGTTGCACAATCAG	pBR-pLac-*orzO* 8(1)9 PCR
EF999	CTGATTGTGCAACATTT**G**GTACCAACGACGTCAG	pBR-pLac-*orzO* 8(1)9 PCR
EF976	GACGTCGTTGGTACGA**C**ATGTTGCACAATCAGCAAG	pBR-pLac-*orzO* 10(1)7 PCR
EF977	CTTGCTGATTGTGCAACAT**G**TCGTACCAACGACGTC	pBR-pLac-*orzO* 10(1)7 PCR
EF745	CTGACGTCGTTGGTACG**C**AATGTTGCACAATCAGC	pBR-pLac-*orzO* 9(1)8 PCR
EF746	GCTGATTGTGCAACATT**G**CGTACCAACGACGTCAG	pBR-pLac-*orzO* 9(1)8 PCR
EF853	GGTTATAATTTA GACGTC GGTACGAAATGTTGCACAATC	pBR-pLac-*orzO*Δ3 PCR
EF875	GGTTATAATTTATTC GACGTC ACGAAATGTTGCACAATC	pBR-pLac-*orzO*Δ6 PCR
EF940	GATACTGACGTCGTTGG**AT**CGAAATGTTGCACAATCAGC	pBR-pLac-*orzO* 5(2)11 PCR
EF941	GCTGATTGTGCAACATTTCG**AT**CCAACGACGTCAGTATC	pBR-pLac-*orzO* 5(2)11 PCR
EF934	GATACTGACGTCGTTGGT**T**CGAAATGTTGCACAATCAGC	pBR-pLac-*orzO* 6(1)11 PCR
EF935	GCTGATTGTGCAACATTTCG**A**ACCAACGACGTCAGTATC	pBR-pLac-*orzO* 6(1)11 PCR
EF972	CGTCGTTGGTTCGAAATGTTG**A**CAATCAGCAAGTTAGCG	pBR-pLac-*orzO* 6(1)10(1) PCR
EF973	CGCTAACTTGCTGATTG**T**CAACATTTCGAACCAACGACG	pBR-pLac-*orzO* 6(1)10(1) PCR
EF944	GATACTGACGTC**C**TTGGTTCGAAATGTTGCACAATCAGC	pBR-pLac-*orzO* 1(5)1(11) PCR
EF945	GCTGATTGTGCAACATTTCGAACCAA**G**GACGTCAGTATC	pBR-pLac-*orzO* 1(5)1(11) PCR

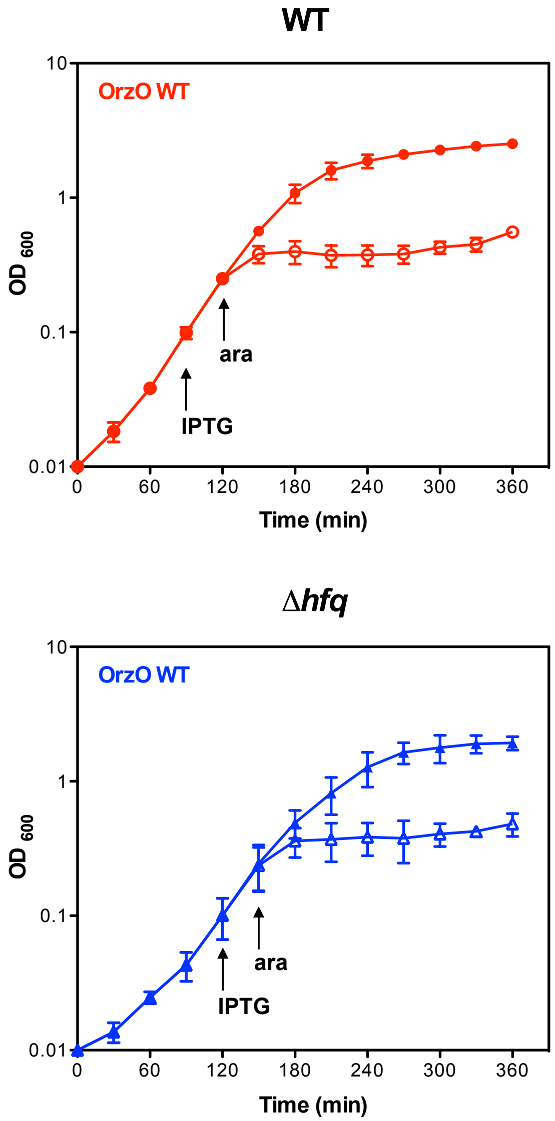
WT
OrzO WT
OD 600
10
1
0.1
0.01
ara
IPTG
0
60
120
180
240
300
360
Time (min)
Δhfq
OrzO WT
OD 600
10
1
0.1
0.01
ara
IPTG
0
60
120
180
240
300
360
Time (min)

Figure S1

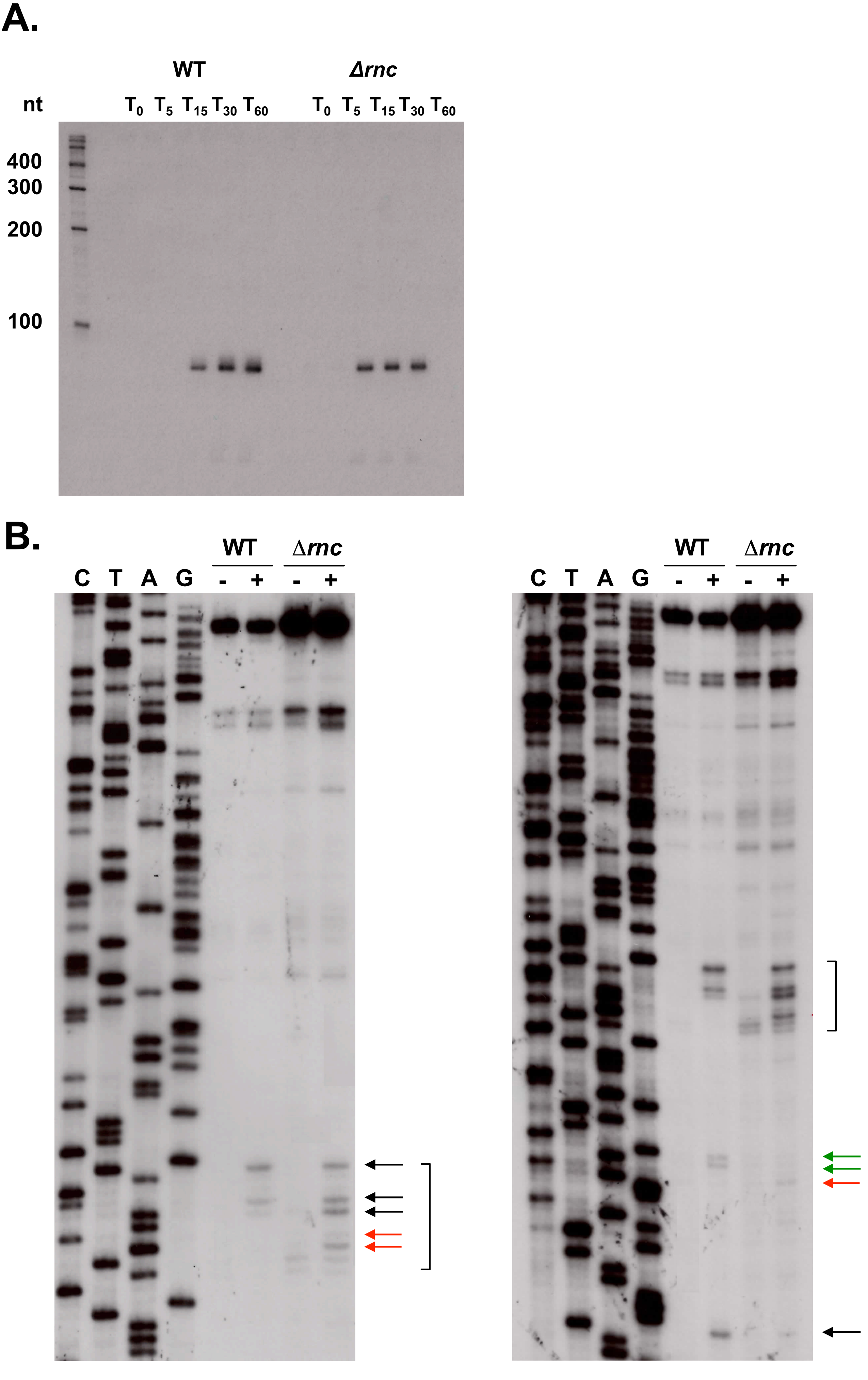

A.
WT
Δrnc
nt
T0 T5 T15 T30 T60
T0 T5 T15 T30 T60
400
300
200
100
B.
WT
Δrnc
C T A G - + - +
WT
Δrnc
C T A G - + - +

Figure S2

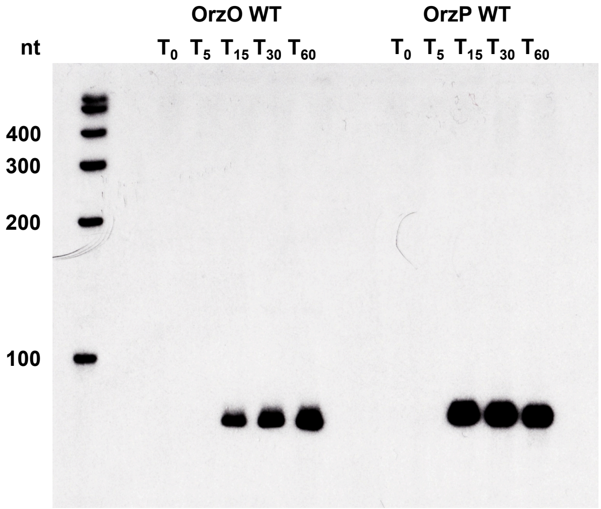
OrzO WT
OrzP WT
nt
T0 T5 T15 T30 T60
T0 T5 T15 T30 T60
400
300
200
100

Figure S3

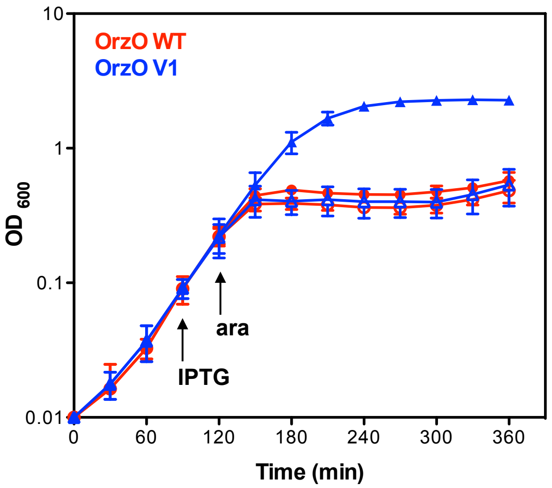


```
OrzO V1 5′ GUUGGUACACGAUGUUGC 3′
           ||||||||||||:|||||
zorO V1 3′ CAACCAUGUGCUGCAACG 5′
```

Figure S4

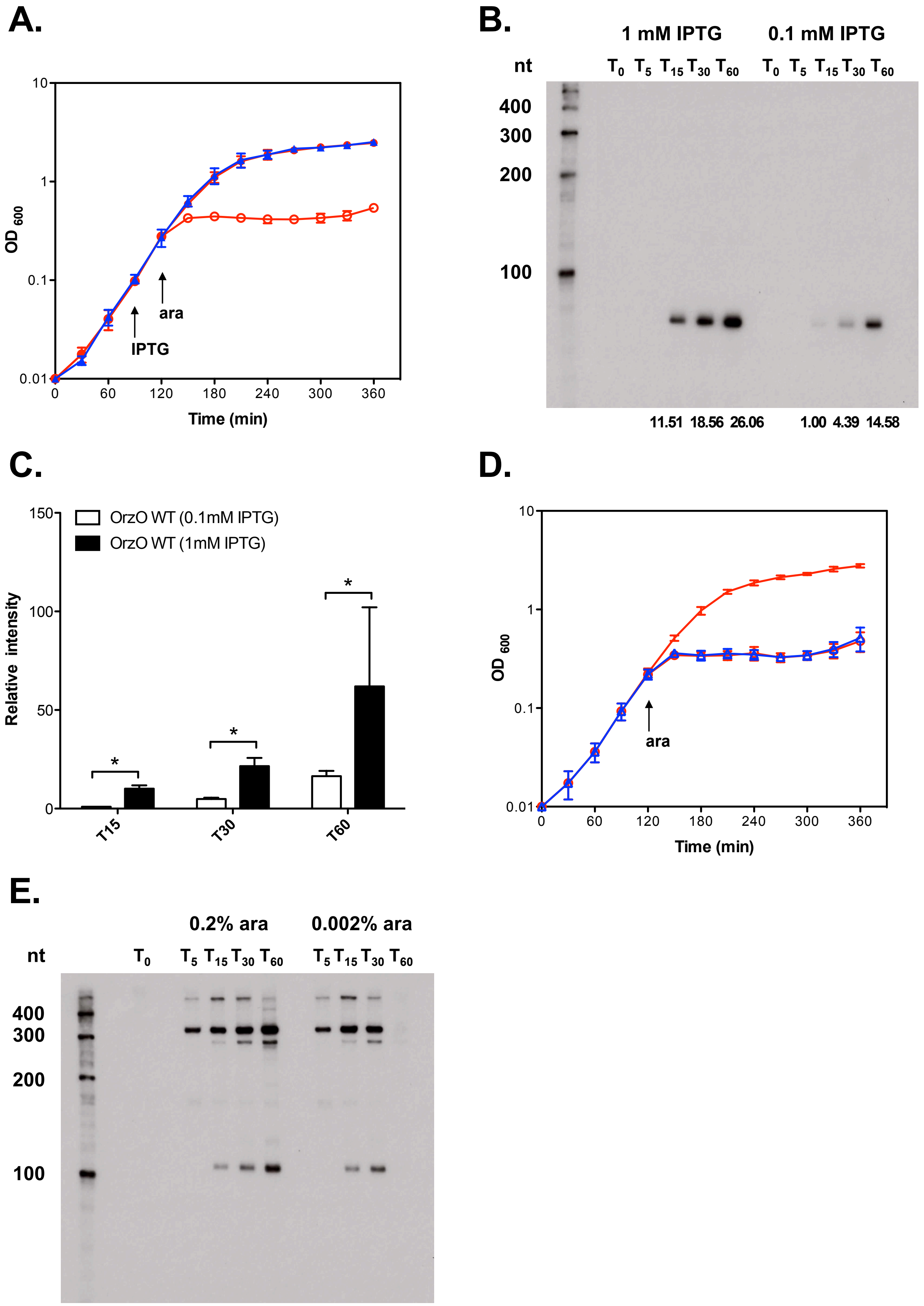
A.
OD 600
Time (min)
IPTG
ara
B.
1 mM IPTG
0.1 mM IPTG
nt
T0 T5 T15 T30 T60
T0 T5 T15 T30 T60
400
300
200
100
11.51 18.56 26.06
1.00 4.39 14.58
C.
OrzO WT (0.1mM IPTG)
OrzO WT (1mM IPTG)
Relative intensity
T15
T30
T60
D.
OD 600
Time (min)
ara
E.
0.2% ara
0.002% ara
nt
T0
T5 T15 T30 T60
T5 T15 T30 T60
400
300
200
100

Figure S5

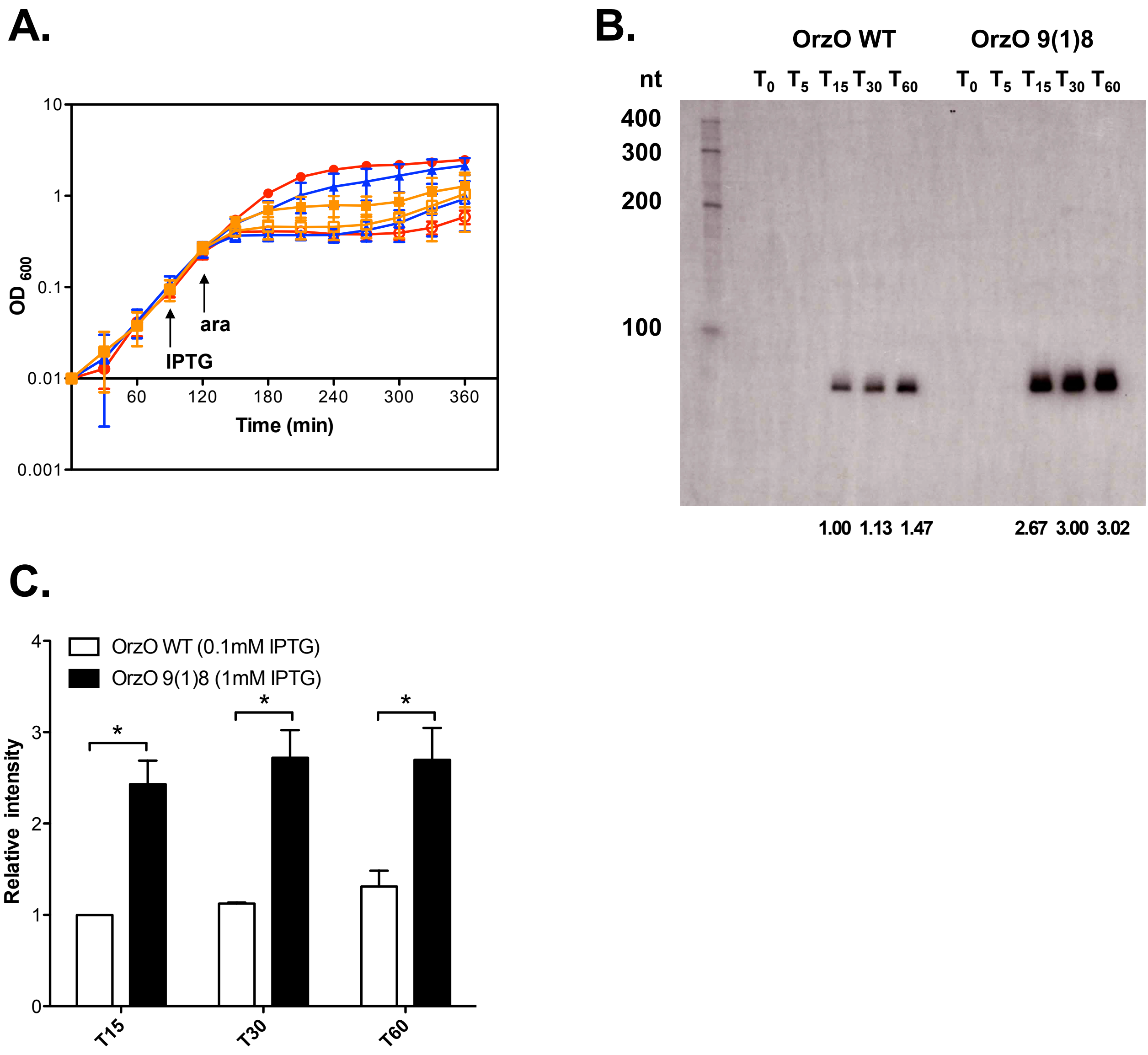
A.
OD 600
10
1
0.1
0.01
0.001
60
120
180
240
300
360
Time (min)
IPTG
ara
B.
OrzO WT
OrzO 9(1)8
nt
400
300
200
100
1.00 1.13 1.47
2.67 3.00 3.02
C.
OrzO WT (0.1mM IPTG)
OrzO 9(1)8 (1mM IPTG)
Relative intensity
T15
T30
T60

Figure S6

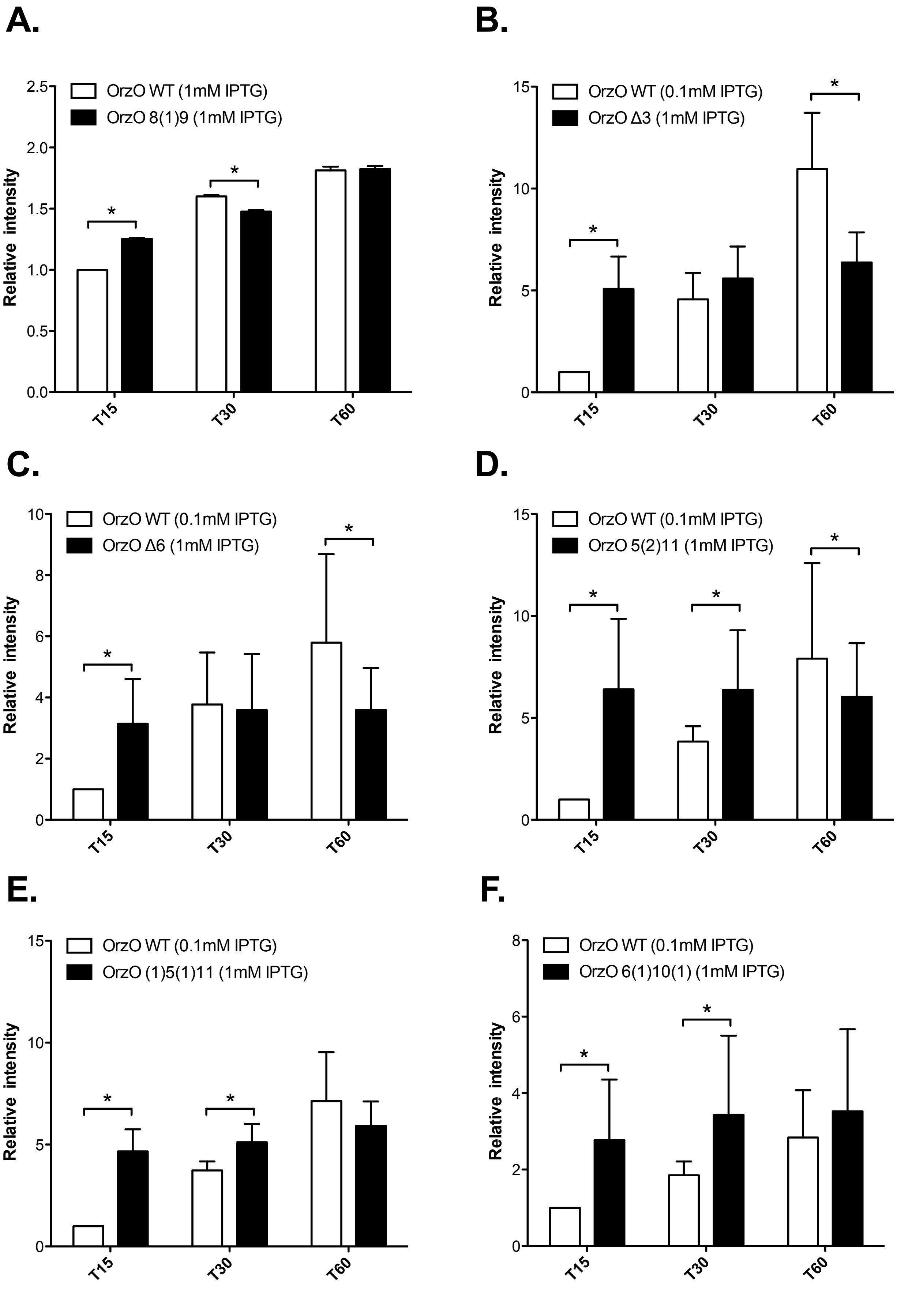
A.
OrzO WT (1mM IPTG)
OrzO 8(1)9 (1mM IPTG)
Relative intensity
0.0
0.5
1.0
1.5
2.0
2.5
*
*
T15
T30
T60
B.
OrzO WT (0.1mM IPTG)
OrzO Δ3 (1mM IPTG)
Relative intensity
0
5
10
15
*
*
T15
T30
T60
C.
OrzO WT (0.1mM IPTG)
OrzO Δ6 (1mM IPTG)
Relative intensity
0
2
4
6
8
10
*
*
T15
T30
T60
D.
OrzO WT (0.1mM IPTG)
OrzO 5(2)11 (1mM IPTG)
Relative intensity
0
5
10
15
*
*
*
T15
T30
T60
E.
OrzO WT (0.1mM IPTG)
OrzO (1)5(1)11 (1mM IPTG)
Relative intensity
0
5
10
15
*
*
T15
T30
T60
F.
OrzO WT (0.1mM IPTG)
OrzO 6(1)10(1) (1mM IPTG)
Relative intensity
0
2
4
6
8
*
*
T15
T30
T60

Figure S7
